# Sport during the COVID-19 bio-bubble: Wellness and opinions in South African elite football

**DOI:** 10.17159/2078-516X/2022/v34i1a12528

**Published:** 2022-01-01

**Authors:** K Bahdur, L Pillay, D Dell’oca

**Affiliations:** 1 Human Movement Science, Faculty of Health Science, Nelson Mandela University, Gqeberha, South Africa; 2 Wits Institute of Sports Health, Department of Health Sciences, University of Witwatersrand, Johannesburg, South Africa; 3 Section Sports Medicine, University of Pretoria, Pretoria, South Africa; 4 Supersport International, Blairgowrie, Randburg, South Africa

**Keywords:** isolation, sport, biologically safe environment, coping mechanisms, mental toughness

## Abstract

**Background:**

COVID-19 imposed challenges on professional sport, with restrictions leading to the delay in the completion of the South African Premier Soccer League (PSL). Creating a biologically safe environment (BSE) provided a solution enabling the 2019/2020 season to be completed.

**Objectives:**

Evaluating the impact that the BSE had on player wellness and what coping mechanisms were used in the BSE.

**Methods:**

A questionnaire was distributed to PSL teams on the final weekend in the BSE. It consisted of three validated psychology questionnaires. An additional section focused on the impact and coping strategies during the PSL’s BSE.

**Results:**

A total of 37 completed questionnaires were analysed. General anxiety (4.7±4.2) and depression levels (4.8±3.9) were at an overall low. The health of the players, as well as separation from and concerns about family, were the greatest contributors to anxiety. Electronic communication with family and friends, social interactions with others in the BSE and time spent on self-reflection were important coping mechanisms for players. As time progressed, they adapted to the BSE.

**Conclusion:**

The BSE did not have a negative impact on the anxiety and depression levels of the respondents, with a variety of coping mechanisms key helping them adapt in the BSE.

The COVID-19 pandemic imposed unique and unfamiliar stress on different population groups, including sporting codes.^[1,2]^ Sports bodies were forced to end the 2019/2020 season without declaring champions (for example, in the Netherlands), or with declaring the top team in the league as champions despite not all matches being played (as in Scotland), or to identify methods to complete their seasons.^[3]^ Most sports opted to complete their 2019/2020 season under restricted conditions, which varied from frequent testing to complete bio-bubbles (i.e. an environment sealed off from the outside world and accessed by a limited number of people only, used to allow events such as sports matches to take place during the coronavirus). The South African Premier Soccer League (PSL) opted for a biologically safe environment (BSE)^[4]^ with all teams and other persons entering the BSE required to be tested for COVID-19 using polymerase chain reaction (PCR) nasal swabs prior to entry to the BSE No contact was made with those outside the BSE and all matches were played in a single province. This meant that all teams, match officials, transport services, media and hotel staff were required to stay in hotels and were faced with other restrictions, including limited contact with teammates and other persons outside of training and matches. Strict schedules dictated movements within the hotels and the BSE environment.^[5]^ All persons within the BSE were subjected to daily symptom and temperature screening at various points in order to ‘red flag’ suspicious cases and to investigate these accordingly. In addition to exposure to pandemic-related stressors, the BSE also increased the risk of magnification of other stressors, e.g. compacted competition loads and limited recovery.

Research has identified different factors as contributors to mental fatigue and depression in football. These included the inability to rest from the sport, lack of job security^[6]^, feeling isolated, lack of time with family and friends, pressure for results, and compacted competition schedules.^[7]^ In addition, long camps and the only interaction within the teams’ football circles can be psychologically stressful, and increased the risk of isolation and decreased motivation. Recent studies of South African athletes during lockdown (Alert Level 5) have shown the physical and mental effects athletes described.^[2]^ Such conditions are likely to induce both mental and physical fatigue and could result in players being at an increased risk of injury.^[8]^ The COVID-19 pandemic has increased symptoms of depression and anxiety, including sleep and mood disturbances, in footballers.^[9]^ BSE restrictions ran the risk of amplifying these factors as players faced isolation, even from teammates. Also, the financial impact of COVID-19 on clubs was unpredictable, resulting in a greater fear of loss of jobs and pay cuts. On the field, pressure to complete the 2019/2020 season with enough time to begin the 2020/2021 season meant matches every two-three days, requiring all personnel to be able to rest from one match and focus on the next, while correcting and adapting strategies and weaknesses from the previous match, in preparation for the next match. This added greater physical and mental strain for all stakeholders.

Normally, players will develop strategies to cope with these challenges. Regular contact with family and social support structures are influential. The BSE restricted such contact with people outside this environment to video, text or make online communication with hotel-limited bandwidth, and personal circumstances leading to data limitation. Usually, over time, players also develop basic pre- and post-match strategies that get them into the right disposition, physically and mentally, for competition. This can include going out with family and/or friends to decompress after a match, or socialising around the hotel pool with specific teammates on game day. The strict guidelines around matches in the COVID-19 bio-bubble meant adjustments had to be made to routines. Spectators also can play a role in motivating teams. Playing behind closed doors (spectatorless) became the norm for all matches. In training, players are exposed to competition-like situations. Coaches plan for a gradual adaptation to new scenarios. The lack of preparation time between matches meant that for many teams competition was coupled with the need to adjust to the ‘new normal’ (i.e. spectatorless and limited physical interactions) and the development of new pre- and post-match habits.^[9]^ The build-up to the resumption of the League also contributes greatly to the emotional and psychological strain.^[9]^

In the BSE, personnel were exposed to an environment which may have added to their psychological load. The lack of evidence-based solutions and prior experience with similar circumstances magnified the challenges experienced. This study examined how living in the BSE impacted on the psychological measures related to anxiety and depression and identified certain strategies that personnel used to cope.

## Methods

### Participants

A total of 37 completed questionnaires were analysed. Respondents were from different clubs in the PSL and to ensure anonymity no identifying characteristics, such as club or age, were recorded. They included analysts (n=2), coaches (n=6), managers (n=4), medical personnel (n=2) and players (n=23). Four players who did not answer the questionnaire from one club were interviewed about their experience in the BSE six months after the BSE. The interviews followed a semi-structured format and were conducted telephonically or virtually. All respondents were male.

### Ethical approval and data collection

The Research Ethics Committee from the Nelson Mandela University applied and was granted ethics approval (Ethics number: H20-HEA-HMS-006). The questionnaire and information sheet were circulated through contacts within the football fraternity in the BSE. Data collection utilising the questionnaire took place during the final weekend of the BSE and interviews were conducted six months after the BSE.

### Instrument

An online questionnaire was developed using a web-based survey tool (https://www.survey monkey.com) to allow an investigation into the health and anxiety profiles, and coping strategies used during the PSL’s BSE. The questionnaire consisted of three standardised psychology questionnaires, and an additional section focused on the impact and coping strategies in the BSE. These were adapted from validated questionnaires, as none existed at the time specific to these scenarios. The questionnaire was organised into four sections:

Section A: General Anxiety Disorder-7 (GAD-7);
Section B: Patient Health Questionnaire-9 (PHQ-9)
Section C: Mental Toughness Inventory -7 (MTI-7)
Section D: Causes of anxiety, impact of the PSL’s BSE and coping mechanisms

The GAD-7 and PHQ-9 were coded as minimal (0–4), mild (5–9), moderate (10–14) and severe (≥15).^[1]^

Interview questions included:

- What was it like playing out the season in the BSE?
- How did it differ from a usual season?
- What did you do to pass the time?
- How did it affect the team?
- Who provided the greatest relief during the BSE?
- How did the team help players cope individually and as a unit?
- How did the break compare to lengthy injury lay-offs?
- How difficult was it to adjust to the bio-bubble?
- How well do you feel the COVID protocols were implemented?
- How did you find not having a roommate impacted you?
- Do you believe the BSE advantaged any teams or players?
- Did you as players feel team morale was severely impacted by not having fans at the venue?

### Statistical analysis

Data were analysed quantitatively and qualitatively as deemed appropriate. Descriptive statistics were tabulated for the categorical and multiple response questions. The Pearson correlation coefficient was used to identify any relationships between mental toughness, general anxiety and the participant’s health. The one-way ANOVA was used to identify any significant differences between the GAD-7, PHQ-9 and MTI based on the identified causes of anxiety and coping strategies utilised. Statistical significance was placed at p<0.05.

## Results

The results are divided into five sections. The first section addresses general anxiety and participant health. The second section focuses on the impact of the BSE and coping strategies used. The third section describes the mental toughness profiles. The fourth section explores relationships between Section 3 and Section 1 and 2. The concluding section summarises the information obtained from the interviews.

### Section 1: Anxiety and participant health

**Fig. 1:**
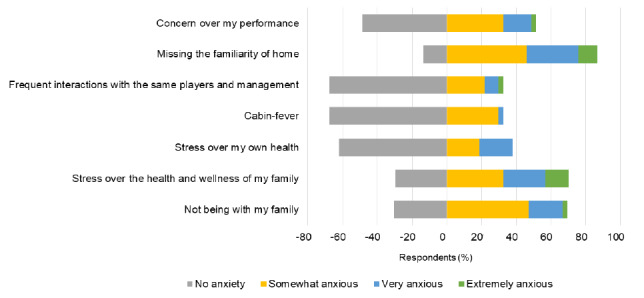
Subjectively identified causes of anxiety for respondents (n=37) using an adapted questionnaire. A negative value indicates no anxiety experienced; a positive value indicates that anxiety was experienced.

GAD-7 results showed low anxiety levels with a mean of 4.7±4.2. Only one respondent had anxiety levels classified as extreme and two others were in the moderate classification. PHQ-9 results were similar with a mean of 4.8±3.9 and none of the results fell into the severe depressive category. Fig. 1 highlights some of the factors contributing to anxiety.

### Section 2: Impact of the BSE

**Fig. 2:**
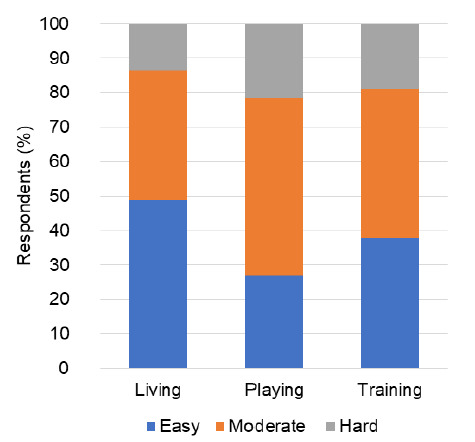
Difficulty experienced by respondents (n=37) in adapting to the biologically safe environment (BSE) under living, training and playing/competition conditions using an adapted questionnaire.

Adapting to the BSE varied according to different participants. Fig. 2 illustrates the breakdown of the difficulty respondents felt when adapting to the BSE in different contexts.

**Fig. 3:**
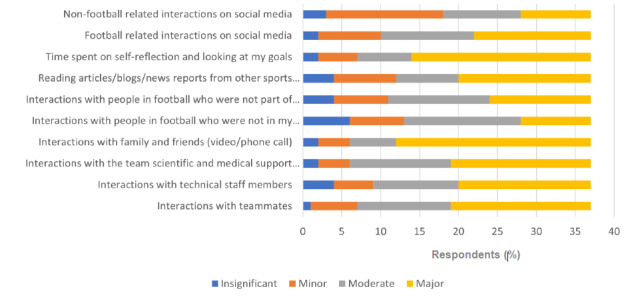
Subjectively identified coping mechanisms and the extent of its effect for respondents (n=37) using an adapted questionnaire. Full column titles: Column 4, “Reading articles/blogs/news reports from other sports people or footballers in other countries”; Column 5, “Interactions with people in football who are not part of the BSE”; Column 6, “Interactions with people in football who are not in my team but subjected to the BSE”; Column 8, “Interactions with the team scientific and medical support structures”.

**Fig. 4:**
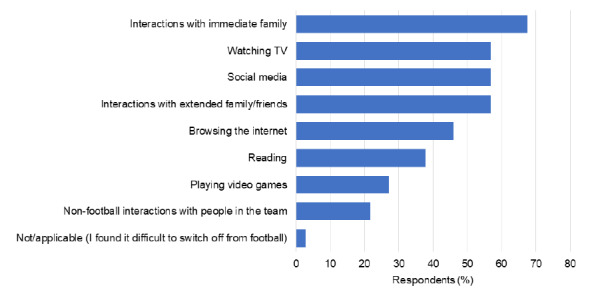
Identified methods to rest from football for respondents (n=37). Methods ranked from most to least effective.

The respondents used different coping mechanisms, which had varying impacts on their ability to adapt to the BSE. Fig. 3 highlights the extent to which different coping mechanisms were effective. Fig. 4 ranks the actions that helped the respondents switch off from football.

### Section 3: Mental toughness

Mental toughness scores within the group were high (47.1±2.1), with only 11% of the respondents scoring less than 20 points in the MTI.

### Section 4: Relationship between Sections 3 and 1

**Fig. 5:**
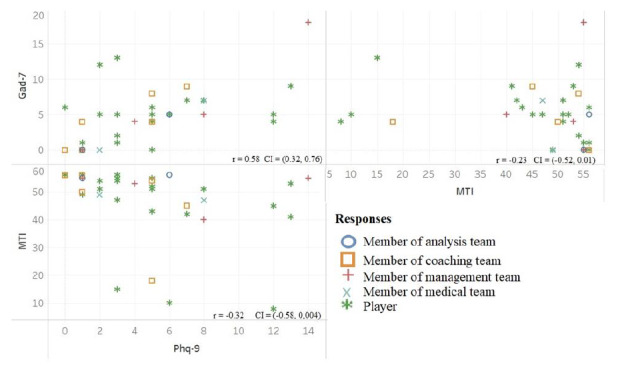
Relationship between General Anxiety Disorder-7 (GAD-7), Mental Toughness Inventory -7 (MTI) and Patient Health Questionnaire-9 (PHQ-9) based on each role played in the team. Team consists of analyst (n=2), coach (n=6), manager (n=4), medical personnel (n=2) and players (n=23). Axes represent score values for each questionnaire.

Fig. 5 shows the correlations between the results of the PHQ-9, GAD-7 and the MTI. This has been broken down based on the role of the respondents. Table 1 summarises the significant relationships between the PHQ-9, GAD-7 and MTI with the causes of anxiety and coping techniques utilised.

### Section 5: Interviews

At the six-month interview, the following emerged:

Three of the players indicated that mental toughness was key in helping them to get through the BSE, but one player highlighted that they were just relieved to be able to finish the season and that “the BSE was well organised which made the transition easier”. One player highlighted that “it got more difficult at the end”, but believed he “needed to persist”.

Players had to adapt to spending more time alone than they were used to in camp settings, with the BSE not allowing players to share rooms. Players saw this as a challenge but found there was greater time for self-reflection and introspection. One player highlighted how he used the time to review his goals, and two players (in addition to the 14 from the questionnaire) spent their time reading. Being away from the family was identified as one of the greatest challenges, and players had to rely on technology to stay in touch.

## Discussion

There is evidence that elite football causes high levels of mental and physical stresses.^[10]^ The causes extend beyond just competition requirements. The nature of the game and fixtures results in footballers often travelling with limited family time. Previous studies have also found that professional sports can lead to feelings of isolation, the inability to rest from the sport, a lack of job security and fears over health and safety, are all stressors.^[6]^

This study identified potential off-field causes of anxiety and coping methods used by football personnel in the PSL’s BSE. General anxiety, mental toughness and participant health scores were also recorded. The COVID-19 pandemic enhanced some of these stressors. Prior to the BSE, there was an expectation of increased risk of anxiety and other mental health symptoms. Therefore, team doctors in the BSE were sensitised to this and details were shared regarding remote access to mental health. In the buildup to the NBA bio-bubble, the NBA highlighted potential mental health risks and encouraged teams to utilise their mental health expert(s).^[11]^

Despite the additional stressors, the overall general anxiety and participant health profiles were good. The BSE served the purpose of reducing the risk of contracting COVID-19. The safety measures and overall organisation put in place could have also contributed to a decrease in anxiety. Also, football is a contact sport, and while measures to enforce social distancing away from training and matches and prior to the start kick-off, during the match, contact between players is inevitable once a match begins. Access to the BSE was allowed following a negative COVID-19 PCR test, and physical interactions were limited to personnel within the BSE who were all subjected to daily symptom screenings.

The COVID-19 bio-bubble did prove effective in controlling the spread of the virus, with only one reported case in the BSE amongst players and one reported case amongst hotel staff. This was similar to the results found in the NBA and WNBA. In other countries and sporting codes without strict restrictions, there were several cases of COVID-19.^[11]^ Players found it challenging to adapt to the conditions.

The GAD-7 and PHQ-9 scores in this context did indicate that psychological distress based on general anxiety and depression was lower in this cohort than in the general population^[12]^. To date, there is limited published research on the effects of BSEs, similar or other contingencies used in professional sport to continue with competition amidst the pandemic. Players have spoken about their expectations and experiences. These did vary with the results of this study. NBA players have identified increased depression and anxiety while being in the NBA bio-bubble.^[11]^

Concerns regarding job security and financial implications were also a cause for anxiety. During the initial stages of the COVID-19 outbreak, there was uncertainty as to whether the 2019/2020 football season would be completed. Club owners and economists expressed concerns regarding the predicted negative financial impacts in general and what people within the professional sports arena were facing.^[13]^ The completion of the season did minimise the possibility of loss of income. Securing salaries of team members could have reduced anxiety.

Mental toughness has been linked with the ability to predict the results on performance sports.^[14]^ From a psychological perspective, mental toughness is the psychological edge that enables athletes to cope with sporting demands and maintain a consistent and higher level of performance than other opponents with determination, confidence and control in high-pressure situations.^[14]^ COVID-19 created a challenging and high-pressured environment related to sport. Duplication of what is stated above. This study found that players with the highest scores in mental toughness found it easiest to adjust under the BSE conditions.

Swedish national female footballers displayed high levels of mental toughness and low anxiety levels when compared to players competing at lower levels.^[15]^ This study found no significant relationship between MT and GAD-7 or PHQ-9 scores, which was contrary to the findings of Bohannan which found weak but significant correlations with PHQ-9 (*r* = −0.318; *p* =0.019) and GAD-7 (*r* = −0.315; *p* = 0.020)^[16]^.

Some of the identified stressors were fixture congestion, having nothing to do all day, time away from family and friends, concerns about safety and the ability to cope with the pandemic.^[11]^ Noted was the importance of social interactions in the team setting. The greatest causes of anxiety was the fear for the health and safety of family members and, as previously mentioned, missing family members. The effect of isolation from family could have been worsened by the fact that prior to the BSE, respondents would have been under harder lockdown rules with their families. Thus, the transition to their absence was even greater.^[17]^ However, people involved in sport at this level are used to going for long periods without physical interaction with families. This is particularly true in cases where clubs are based in different cities or countries.^[7]^

Mental health experts suggested maintaining social connections and interactions, including non-football interactions within the team set-up and partaking in leisure activities, such as video games, as possible ways to cope with being in the bio-bubble. This study did find these to be useful tools. The most significant coping strategy was linked to social interactions with family and friends, then social media, and lastly, teammates.^[17]^ While interactions with team members and other personnel in the BSE were identified as a coping technique, there was some evidence that spending too much time with them contributed to higher GAD-7 scores. Other contributors to anxiety included cabin-fever, which was similar to findings in the general population where people were confined within their homes.

Respondents reported on ways in which they rested from football. Staying in touch with family and friends was the most utilised tool, followed by social media, and watching television. The use of social media as a means of resting from football is an unexpected finding, since it can increase a player’s exposure to football-related information directly related to them or their team, or also unrelated football content. Constant exposure to social media increased the exposure to COVID-19-related news and anxieties related to other social media users.

The inability to take a break from football is a contributor to mental fatigue and is therefore vital for the physical and mental recovery of players.^[6]^ The PSL’s BSE differed from the NBA where an entire resort was utilised. As previously mentioned, there was access to entertainment and leisure activities which created opportunities to take a break from the game. Close family and friends were also allowed to join the bio-bubble in the latter stages. The length of the PSL’s BSE was shorter. Teams were based at different hotels and allocated different training grounds and stadiums for the duration of the BSE. Access to other activities was limited according to hotel amenities.

Self-awareness using self-reflection has a positive association with mental toughness (MT) in tennis players.^[18]^ In this study, there was no relationship between MT and self-reflection as a coping method. However, self-reflection, combined with examining the respondents’ goals, was a significant coping mechanism used in the BSE. Introspection was also identified as a tool for cricketers facing BSE situations. The possible negative emotions linked to extensive time with one’s thoughts was highlighted as a risk factor for negative emotions.^[19]^

The advantages of completing the season and the contribution of the BSE that enabled the completion of the season, helped mitigate possible negative effects of being in the BSE.

### Limitations

The cohort was small – only 37 respondents. There were no pre-COVID-19 profiles, thus it was not possible to see how COVID-19 impacted on the psychological profiles. The positive GAD-7 and PHQ-9 scores could be related to the timing of the data collection. Teams were in the BSE for six weeks, and the GAD-7 and PHQ-9 were completed at the end of the BSE. By this time, players were used to the environment and with the end now close, it could have made them feel better.

Self-reported tools of assessment can sometimes lead to biased responses with respondents opting to provide the responses they feel are expected of them and likely to make them appear more favourable. This has been identified as a reason for the miscalculation of the occurrence of depression.^[20]^ The use of anonymous surveys, with limited identifying questions, was undertaken to minimise the effect of measured responses in the questionnaire.

## Conclusion

Despite this being the first exposure to a BSE, the report of anxiety was low, with no evidence of a reduced mood within the BSE. The utilisation of different coping mechanisms and tools to rest from football, as well as the use of technology to keep in touch with people outside the BSE, and the support and interactions with team personnel contributed to making the BSE easier to manage. Mental toughness and resilience, which are highlighted as important characteristics for success in elite sport, could contribute to the adaptations to the changing circumstances.

## Figures and Tables

**Table 1:** The significant relationship between GAD-7, MTI and PHQ-9 with causes of anxiety and coping mechanisms

Questionnaire	Impact, cause of anxiety or coping mechanism	p-value
**GAD-7**	Anxiety: Concern over my performance	0.005
	Anxiety: Frequent interactions with the team	0.004
	Anxiety: Cabin-fever	0.000
	Anxiety: Being away from family	0.005

**PHQ-9**	Anxiety: Being away from family	0.018
	Anxiety: Stress over health of family	0.010
	Anxiety: Frequent interactions within the team	0.000
	Anxiety: Missing familiarity of home	0.001
	Coping: Interactions with technical team	0.017
	Coping: Interactions with people from other teams who were in the bio-bubble	0.046
	Playing	0.012
	Training	0.024

**MTI**	Training	0.024
	Living	0.035
	Coping: Interactions with family and friends	0.011
	Coping: Interactions with scientific and medical team	0.041
	Anxiety: Health and wellbeing of family and friends	0.030

GAD-7, General Anxiety Disorder-7; PHQ-9, Patient Health Questionnaire-9; MTI, Mental Toughness Inventory -7.
